# Toxic Metals (Al, Cd, Pb) and Trace Element (B, Ba, Co, Cu, Cr, Fe, Li, Mn, Mo, Ni, Sr, V, Zn) Levels in *Sarpa Salpa* from the North-Eastern Atlantic Ocean Region

**DOI:** 10.3390/ijerph17197212

**Published:** 2020-10-02

**Authors:** Alberto Gutiérrez-Ravelo, Ángel J. Gutiérrez, Soraya Paz, Conrado Carrascosa-Iruzubieta, Dailos González-Weller, José M. Caballero, Consuelo Revert, Carmen Rubio, Arturo Hardisson

**Affiliations:** 1 Toxicology Area, Universidad de La Laguna, La Laguna, 38071 Santa Cruz de Tenerife, Spain; albertogutierrezrv@gmail.com (A.G.-R.); spazmont@ull.edu.es (S.P.); dgonzal@ull.edu.es (D.G.-W.); jcabmes@hotmail.com (J.M.C.); crubio@ull.edu.es (C.R.); atorre@ull.edu.es (A.H.); 2 Department of Animal Pathology and Production, Bromatology and Food Technology, Faculty of Veterinary, Universidad de Las Palmas de Gran Canaria, 35001 Arucas, Spain; conrado.carrascosa@ulpgc.es; 3 Canarian Public Health Service, Central Laboratory, 38006 Santa Cruz de Tenerife, Spain; 4 Department for Physical Medicine and Pharmacology, Universidad de La Laguna, La Laguna, 38071 Santa Cruz de Tenerife, Spain; mgirones@ull.edu.es

**Keywords:** toxic metals, trace elements, marine pollution, risk assessment

## Abstract

*Sarpa salpa* is a fish belonging to the Sparidae family and is usually found in local markets. Toxic metals such as aluminum (Al), cadmium (Cd), lead (Pb) and trace elements such as boron (B), barium (Ba), cobalt (Co), copper (Cu), chromium (Cr), iron (Fe), lithium (Li), manganese (Mn), molybdenum (Mo), nickel (Ni), strontium (Sr), vanadium (V) and zinc (Zn) are incorporated into fish tissues and remain there. The liver has the highest concentrations of all the analyzed toxic metals and almost all the analyzed trace elements. The consumption of 100 g/day of *S. salpa* muscle tissue does not pose a health risk. However, 100 g/day of liver consumption may pose a serious health risk due to the intake of Cd (572% of the tolerable weekly intake for adults with a body weight of 68.48 kg) and Pb (117% of the tolerable daily intake for adults weighing 68.48 kg). The consumption of liver of this species is not recommended due to its possible harmful effects on health.

## 1. Introduction

Salema or *Sarpa salpa* is a fish belonging to the Sparidae family. This species is common on the coasts of the Canary Islands and is normally available in local markets. The salema is an elongated and oval-shaped fish, whose length can reach 50 cm. This fish lives near the coasts and forms coordinated schools/shoals to evade predators. It is one of the few herbivorous fish that can be found in the Canary Islands, and it mainly feeds on algae [1]. This specie lives in the beach line and shows territorial lifestyle habits. Normally, the salema lives in areas with abundant vegetation and algae. Salema is found at depths of from 1 to 30 m [1].

Tenerife is the island with the largest surface area (2034.38 km^2^) of the Canary Archipelago and is also the most populated island (904,713 inhabitants), with a population density of 445 inhabitants/km^2^ [2,3]. Coupled with the large permanent population of the island is the large influx of tourists, meaning that the island of Tenerife is subject to marked anthropogenic pollution, such as the effluents from sewage outlets, the transit of large cruise ships, merchant ships and oil tankers, etc. [4,5].

Marine pollution is increasing and has a notable impact on human nutrition, since some of the pollutants found in the marine environment could be absorbed by fish, mollusks, crustaceans, and algae. Although marine pollutants are highly varied, toxic metals and trace elements are noteworthy for their persistence and easy incorporation into animal and plant tissues [6,7,8].

Toxic metals such as aluminum (Al), cadmium (Cd) or lead (Pb) are characterized by their high half-life. The high intake of Al has been related to neurodegenerative diseases such as Alzheimer’s disease [3,9,10]. Furthermore this element can interfere with essential elements such as calcium (Ca) [11,12]. Cd is a well-known nephrotoxic agent which can also cause damage to the central nervous system, alterations in sexual reproduction and even infertility, psychological disorders, etc. [13,14,15]. High Pb intakes can cause high blood pressure, memory problems, kidney disease, and disorders of the gastrointestinal tract [16,17]. Furthermore, Alzheimer’s is associated with Pb exposure [16,17]. Al, Cd and Pb are considered among the worst pollutants in aquatic systems [18].

As for the trace elements such as boron (B), barium (Ba), cobalt (Co), copper (Cu), chromium (Cr), iron (Fe), lithium (Li), manganese (Mn), molybdenum (Mo), nickel (Ni), strontium (Sr), vanadium (V) and zinc (Zn), they are found naturally in the marine environment. Not only are they essential for certain vegetables and animal organisms, but they are also toxic when ingested in high amounts. Table 1 summarized the principal adverse effects caused by high intakes of these elements.

Fish are a major food source, but high concentrations of heavy metals and trace minerals create a potential health risk [29]. Specific population groups, such as pregnant women, children, or large consumers, are more susceptible to the potential adverse effects of toxic metals and trace elements.

The fish consumption of the Canary Islands’ population in 2013 was 49.9 g/day [30]. Salema is an abundant fish on the coasts of the Canary Islands. It is necessary to take into consideration that the importance of studying the content of metals and trace elements in this species lies in the fact that it is one of the most fished and consumed species by the Canary Islands’ population. Salemas are usually obtained from self-fishing. Salema is a blue fish with a very strong flavor. Generally, the salema is consumed as fillet. The liver of salema is also consumed by humans. The fish liver is sold in cans and considered beneficial to health. This fish is frequently found in local markets and restaurants of the islands due to its culinary properties, hence, it is necessary to determine the level of these elements and to subsequently evaluate the risk.

The aims of this work were (i) to determine the content of toxic metals (Al, Cd, Pb) and trace elements (B, Ba, Co, Cu, Cr, Fe, Li, Mn, Mo, Ni, Sr, V, Zn) in *Sarpa salpa* caught in coastal waters around the island of Tenerife (Canary Islands, Spain); (ii) to compare the content of toxic metals and trace elements between the different collection points and between different tissues (muscle vs. liver tissue); (iii) to study possible correlations between weight, size and concentration of toxic metals and trace elements; and (iv) to calculate the dietary intake of these elements from the consumption of this fish and assess the possible toxic risk.

## 2. Material and Methods

### 2.1. Sampling Area

**Figure 1:**
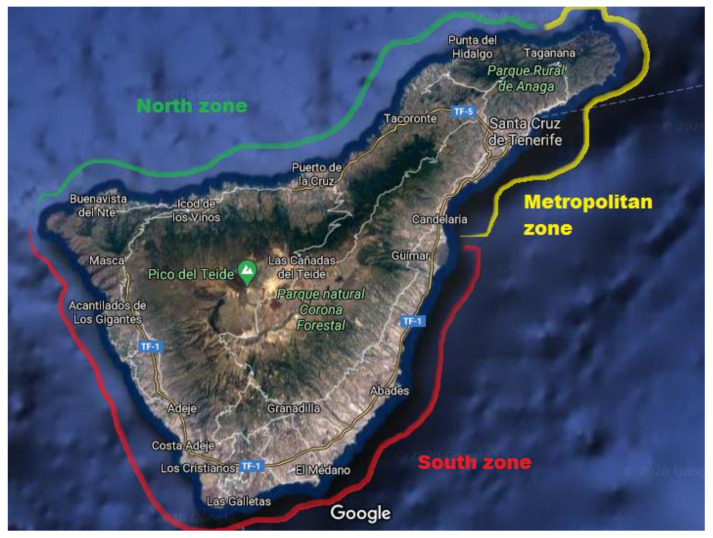
Sampling zones around the island of Tenerife.

The island of Tenerife (28.1723° N, 16.3720° W), located in the northeast of the Atlantic Ocean, was divided into three sampling areas for the purposes of the study (Figure 1), representing the entire coastline of the Tenerife island.

- Metropolitan zone: this covers the area from Punta de Anaga (Anaga Rural Park, Santa Cruz de Tenerife) to Candelaria. The average weight of the specimens (*n* = 10) is 93.7 ± 27.1 g, and their average length is 18.7 ± 1.83 cm.
- South zone: this covers the area from El Médano (Granadilla de Abona) to Punta de Teno (Teno Rural Park, Buenavista del Norte). The average weight of the specimens (*n* = 10) is 234 ± 35.5 g, and their average length is 24.8 ± 1.87 cm.
- North zone: this covers the area from Punta de Teno (Teno Rural Park, Buenavista del Norte) to Punta de Anaga (Anaga Rural Park, Santa Cruz de Tenerife). The average weight of the specimens (*n* = 10) is 233 ± 52.0 g, and their average length is 24.1 ± 1.64 cm.

The different areas were chosen based on geographic factors: the south zone is the most touristic area of the island; the north zone is the area with the strongest waves and currents, as well as being an area with abrupt relief; and the metropolitan area, including Santa Cruz and Candelaria, is the most industrialized area, where the port of Santa Cruz de Tenerife is located (place of transit for large cruise ships, oil and cargo ships, etc.). The thermal power station of Caletillas is located in the city of Candelaria. 

A total of 30 specimens (10 specimens per sampling area) were analyzed. The specimens selected for sampling were acquired from the fishermen’s organization in the previously referenced areas. This study is supported by the fact that this species shows territorial lifestyle habits. Salema is a fish that lives in areas near the coast.

### 2.2. Sample Treatment

The laboratory material was previously washed with Milli-Q quality distilled water (Milli-Q water purification system, Millipore, Burlington, MA, USA) and laboratory detergent (Acationox, Merck, Darmstadt, Germany). Only analytical quality reagents were used.

Samples weighing 10 g of muscle mass with the skin from the mid-dorsal musculature area were weighed separately. The whole liver of each specimen of variable weight depending was also weighed. The samples were placed in porcelain capsules (Staatlich, Werheim, Germany) and left at a temperature of 70 ± 10 °C for 24 h in an oven (Nabertherm, Lilienthal, Germany) for complete drying. Subsequently, they were subjected to acid digestion with 65% nitric acid (HNO_3_) (Sigma Aldrich, Steinheim, Germany), and, once all the acid had evaporated, they were placed in a muffle furnace (Nabertherm, Lilienthal, Germany) with a temperature–time program of 420 ± 20 °C–24 h, with a progressive rise in temperature of 50 °C per hour [2,31]. The ashes obtained were dissolved in 1.5% HNO_3_ solution (Sigma Aldrich, Steinheim, Germany) up to a total volume of 25 mL [2,31]. Finally, they were transferred to sterile and hermetic polyethylene containers for further analysis.

### 2.3. Analytical Method

The content of toxic metals and trace elements was determined by inductively coupled plasma optical emission spectrometry (ICP-OES) (Thermo Fisher Scientific, Waltham, MA, USA) [32,33]. ICP-OES is an analytical technique with high precision and accuracy. The liquid sample is introduced in the nebulizer, where an aerosol is formed, and then it is transported by the argon gas to the plasma torch. In the plasma, the atomization and ionization of the elements occurs, obtaining the atomic spectra of characteristic lines which are dispersed by the diffraction grating. The detector measures the intensities of the lines [34]. The instrumental conditions of the apparatus were the following: approximate RF power (radiofrequency) of 1.2 kW, gas flow (nebulizer flow, auxiliary flow) of 0.5 L/min, injection pump speed of 50 rpm and stabilization time of 0 s. The ICP-OES determination used liquid argon (99.999% purity, Air Liquid, Spain).

The instrumental wavelengths (nm) were: Al (167), B (249.7), Ba (455.4), Cd (226.5), Co (228.6), Cr (267.7), Cu (327.3), Fe (259.9), Li (670.8), Mn (257.6), Mo (202), Ni (231.6), Pb (220.3), Sr (407.7), V (310.2), and Zn (206.2). The instrumental limits of quantification (LOQ) were calculated as 10 times the standard deviation (SD) resulting from the analysis of 15 targets under reproducibility conditions [35] and were as follows: Al (0.0012), B (0.012), Ba (0.005), Cd (0.001), Co (0.002), Cr (0.008), Cu (0.012), Fe (0.009), Li (0.013), Mn (0.008), Mo (0.002), Ni (0.003), Pb (0.001), Sr (0.003), V (0.005), and Zn (0.007).

The quality control of the analytical method is based on the accuracy and precision values from the recovery study (Table 2) conducted using standard reference materials (SRM) from the National Institute of Standards and Technology (NIST). The CRM used were the SRM 1548a typical diet, SRM 1515 apple leaves and SRM 1567a wheat flour. In the case of Li, the method of standard additions was used, adding known quantities of Li to dehydrated samples of the fish (muscle and liver). High recovery percentages (R) of over 98% were found, with no significant differences (*p* < 0.05) between the concentration certified by the manufacturer and the one found here.

### 2.4. Statistical Analysis

IBM Statistics SPSS 24.0 (IBM Corporation Armonk, NY, USA) for Windows™ was used to perform the statistical analysis of the obtained data. The statistical analysis was conducted to study the possible differences between the study zones (metropolitan, south, and north zone) and between tissues (muscle and liver).

Normality of the results was verified by means of the Kolmogorov–Smirnov test as well as the homogeneity of variances, which was studied by means of the Levene test [36]. The obtained data did not follow a normal distribution, and a non-parametric test was used, in this case the Kruskal–Wallis test. Statistically significant differences are considered as *p* < 0.05.

In addition, correlation analysis was performed using the Spearman correlation coefficient with the aim of determining possible correlations between weight, size and concentration of toxic metals and trace elements.

### 2.5. Dietary Intake Calculations

Once the EDI (estimated daily intake) value has been obtained for each toxic metal and trace element, the contribution percentage is obtained. Contribution percentages are calculated as follows:

% Contribution = [EDI/Guide value] 100

The contribution percentage is used to calculate the contribution percentage to the established guide value and to determine whether the risk is high or not.

## 3. Results and Discussion

### 3.1. Concentration of Toxic Metals and Trace Elements in Liver and Muscle Tissue

Table 3 shows the mean concentrations (mg/kg wet weight) and standard deviations (SD) of the toxic metals and trace elements in the liver and muscle of the analyzed specimens, without geographical distinction.

Liver tissue had the highest concentrations of all the toxic metals analyzed and of almost all the trace elements analyzed, except for Cr, whose highest mean concentrations were recorded in the muscle. The toxic metals and trace elements in the muscle tissue were in the following concentration sequence from highest to lowest: Zn > Fe> Al > Ba > Cu > Sr > Mn > Cr > Li > B > Ni > V > Pb > Co > Cd. In the liver tissue, the sequence of toxic metals and trace elements differs from that of the muscle and was as follows: Fe > Zn > Cu > Al > Sr > B > Li > V > Ba > Cd > Mn > Ni > Pb > Co > Mo > Cr. The Canary Islands are under the phenomena of haze dust due to the fact that this archipelago is close to the Sahara Desert. The sand has high Fe, Cu and Zn concentrations that influence the content of Fe, Cu and Zn of marine organisms like fishes.

The liver had noteworthy concentrations of Fe (221 ± 158 mg/kg wet weight). The liver normally tends to accumulate higher content of Fe. Afonso et al. [5] reported concentrations of Fe (532.78 ± 295.63 mg/kg ww) in the liver of *Sarpa salpa* higher than those found in the present study.

The content of Al (36.2 ± 28.9 mg/kg ww) found in liver tissue is also worth mentioning because it is much higher than that recorded in muscle tissue (1.46 ± 0.78 mg Al/kg ww). Other authors have also found higher Al concentrations in liver than in muscle tissue [5].

The mean Cd content (1.40 ± 1.93 mg/kg ww) found here in the liver is notable, being much higher than that observed in the muscle tissue (0.007 ± 0.01 mg Cd/kg ww). Other authors have also found higher concentrations of Cd in fish liver of than in fish muscle [5,8,37]. According to the European Commission Regulation (EC) 1881/2006 of 19 December 2006, the maximum permitted content level of Cd in fish intended for human consumption is 0.05 mg/kg of fish. Therefore, the mean average content level of Cd found in the present study in muscle tissue is below this limit, meaning it is suitable for human consumption [38]. However, considering the above guideline, the liver would not be suitable for consumption.

The concentration of Pb recorded in liver tissue (0.43 ± 0.59 mg Pb/kg ww) is much higher than that found in muscle tissue (0.03 ± 0.01 mg Pb/kg ww). The European Commission regulation (EC) 1881/2006 of 10 December 2006 sets a legal maximum limit of Pb at 0.3 mg/kg of fish [38]. The concentration levels determined here in the muscle are below the maximum permitted limit, while the mean Pb concentration found in the liver tissue considerably exceeds this limit, and, therefore, its consumption is not recommended. These data agree with those obtained by other authors who have reported higher concentrations of Pb in the liver than in muscle tissue [5,39].

### 3.2. Concentration of Toxic Metal and Trace Elements in Each Sampling Zone

Table 4 shows the mean concentrations (mg/kg ww) and standard deviations (SD) of the toxic metals and trace elements in each of the sampling areas.

In muscle tissue, the Zn content (17.4 ± 1.18 mg/kg ww) found in the specimens from the south zone is noteworthy, while in the specimens from the north zone, the mean average level of Fe (12.5 ± 11.1 mg/ g ww) is higher than the Fe level of the south and metropolitan areas. 

The mean level of Al (1.69 ± 1.18 g/kg ww) in the muscle tissue of the specimens from the metropolitan zone is noteworthy. In specimens such as brook trout, muscle tissue is reported to accumulate less Al than other tissues [40], as has been verified in the results obtained in the present study in *Sarpa salpa*.

The statistical study by zones shows the existence of significant differences in muscle tissue (*p* < 0.05) in the Cd content between specimens from the north zone and south zone with specimens from the metropolitan zone; this is likely due to the fact that the specimens from south zone were the biggest (24.8 ± 1.87 cm), indicating that they accumulated high Cd concentrations [29].

The B content was statistically different among the three areas, with the B content being high in specimens from the south zone. Regarding the Co content, there are significant differences (*p* < 0.05) between the north and metropolitan zone with the south zone, while the mean Cu level differs statistically in the north and south zone compared to the metropolitan zone. The V content differs statistically between the metropolitan and south zone with the north zone. These differences are likely due to several factors like different ocean currents, the pollution of each zone, temperature, etc. In liver tissue, the content of Fe (301 ± 181 mg/kg ww), Cu (87.0 ± 112 mg/kg ww) and Al (57.7 ± 30.6 mg/kg ww) in specimens from the metropolitan zone stand out. It should be noted that, in fish, the tissue concentration of Al is related to the quality of the water. Higher concentrations were recorded in the liver in the metropolitan zone, as this is the most industrialized zone, while the mean Zn level (113 ± 105 mg/kg ww) in the specimens from the north zone is notable.

In the case of the Al concentration in the liver, there are significant differences (*p* < 0.05) between the metropolitan zone, where the highest Al (57.7±30.7 mg/kg) level was recorded, and the south and north zones. The metropolitan zone is under anthropic activities because it is the area with the highest population, and it industrialized and a zone with an important transit for large cruise ships, oil and cargo ships. The content of B, Ba and Pb in the south and north zones is statistically different from the content of these trace elements found in the metropolitan zone. The content of Fe and Cr in specimens from the metropolitan zone differ statistically from the content of Fe and Cr found in specimens from the north zone. The highest Fe and Cr concentrations were found in the metropolitan and south zones, respectively, while the Sr and V contents of the specimens caught in the metropolitan zone are statistically different from the content observed in those from the south zone. The highest Sr level was recorded in the metropolitan zone, which is the most polluted zone because of its industrial activity and high-density population. 

It should be noted that the lowest mean concentrations of the analyzed elements in liver tissue were recorded in the north zone specimens. The north zone is under a lower anthropic activity, and the pollution is this area is low as well as the population density and the industrial activities. However, in muscle tissue, the specimens from the north zone had the highest concentrations of elements like Fe, Cr, Cu and B.

The correlation study (Table 5) based on the Spearman correlation coefficient showed a negative correlation between the length and weight of the captured specimens for Pb content. In other words, the greater the size and weight of the specimen, the lower the Pb content in both muscle and liver. It should be noted that Pb can compete with Ca at the binding sites in hydroxyapatite crystals, which are fixed to the bone [39,41]. It was found that, in the largest specimens from the north and south zones, Pb probably is fixed in the bones, while in the metropolitan zone, young specimens had higher concentrations of Pb accumulated in soft tissues (liver and muscle) [39]. The metropolitan zone, located between Anaga and Candelaria, is the most industrialized area, as it includes the Caletillas thermal power station and the port of Santa Cruz de Tenerife, which has a large influx of ships and large vessels. Therefore, the highest concentration of Pb is due to both physiological factors and increased environmental contamination.

On the other hand, the Spearman correlation coefficient showed a positive correlation between length and weight with respect to the concentration of Cd. That is, the greater the size and weight of the specimens, the higher the Cd content. The specimens from the metropolitan zone had lower levels of Cd since they are young specimens of smaller size and weight, while the specimens from the north and south zones, being larger and heavier, had higher Cd concentrations.

### 3.3. Evaluation of Dietary Intake of Toxic Metals and Trace Elements

Table 6 shows the estimated daily intake (EDI) values (mg/day) of the toxic metals and trace elements analyzed and the percentages of contribution to the respective maximum intakes set by various institutions.

In the case of Cd, considering a consumption of 100 g of salema liver per day, the percentage of contribution to the tolerable weekly intake (TWI), established at 2.5 µg/kg body weight/week [42], is much higher than the fixed value, which is 572% for adults with an average weight of 68.48 kg. Likewise, the consumption of 100 g of liver per day represents a percentage of contribution to the tolerable daily intake (TDI) of Pb, set at 0.5 µg/kg body weight/day [41], of 117% for adults weighing 68.48 kg on average. On the other hand, although they are not exceeded, the percentages of contribution to the maximum value (UL) set by the IOM (Institute of Medicine) [21] for Fe (49.1%, adults), Cu (47%, adults) and Mo (40.0%, adults) from the consumption of 100 g/day of liver are significant. Fish liver consumption has health benefits because it is rich in polyunsaturated fatty acids [47]. Brustad et al. [48] studied the vitamin D status in a rural population of northern Norway with high fish liver consumption and concluded that the consumption of fish liver offers a high vitamin D intake. The Chalmers University of Technology research concluded that fatty fish without environmental pollutants protect against type 2 diabetes [49]. However, in the case of our study, salema liver consumption is not recommended as it represents a significant health risk due to its possible toxic effects because of its toxic metal levels. 

On the other hand, the consumption of 100 g of salema muscle per day does not entail any health risk, since the contribution percentages obtained are well below 100% of the maximum value established for both toxic metals and trace elements.

## 4. Conclusions

Metal concentrations in the *Sarpa salpa* species were significantly higher in the liver than in the muscle, except for Cr, whose levels were higher in muscle tissue. In accordance with the guidelines set out in the European Commission Regulation (EC) 1881/2006 of 19 December 2006, the mean levels of Cd and Pb found in the samples analyzed did not exceed the maximum values set in the legislation and are, therefore, fit for consumption. However, the Pb and Cd values found in the liver samples were well above the value set in the regulation, and their consumption is not recommended.

The correlation study revealed a negative correlation between the length and weight of the captured specimens with the content of Pb; that is, the greater the size and weight of the specimen, the lower the Pb content in both muscle and liver tissue. However, a positive correlation was found for Cd, leading to the conclusion that the greater the size and weight of the specimens, the higher the Cd levels.

The evaluation of the dietary exposure shows that the consumption of the muscle tissue of the *Sarpa salpa* does not pose any risk for the health of consumers. Nevertheless, the consumption of liver tissue can pose a serious health risk, because 100 g a day of liver means intakes of Cd and Pb higher than the maximum set values.

## Figures and Tables

**Table 1:** Possible adverse effects caused by high intakes of trace elements.

Element	Effects	Reference
Fe	Tissue damage by the formation of free radicals of active oxygen species	[19,20]
Zn	Interferences in Cu absorption, which can cause serious neurological diseases	[21]
Cu	Liver damage	
Cr	Chronic kidney failure, dermatitis, bronchitis, and asthma	[21,22]
Co	Calcium homeostasis alteration	[21]
Mo	Effects on the reproductive system observed in experimental animals	
Mn	Neurotoxic; effects on the central nervous system; can cause an increase in blood concentration, muscle weakness and affect motor coordination	[21,23]
B	Adverse effects on development and reproductive function in experimental animals	[21]
Ba	Tachycardia, hypertension, hypotension, muscle weakness and paralysis	[24]
V	Gastrointestinal disorders in humans	[21]
Sr	Phosphorus deficiency and an increase in bone density	[25]
Li	Altered consciousness, tremors, ataxia, apathy, nausea, vomiting, polyuria and / or myopathy	[26]
Ni	Effects in individuals with hypersensitivity to Ni or with kidney problems	[21,27,28]

**Table 2:** Recovery study with the standard reference materials (SRM) for the analyzed metals.

Metal	Material	Concentration Found (mg/kg)	Certified Concentration (mg/kg)	R (%)
Al	SRM 1515 Apple Leaves	286 ± 9	285.1 ± 26	99.7
B		27.0 ± 2.0	27.0 ± 1.5	99.9
Cr		0.30 ± 0.00	0.29 ± 0.03	97.8
Mo		0.09 ± 0.01	0.09 ± 0.02	99.4
Sr		25.0 ± 2.0	24.6 ± 4.0	98.3
Ba	SRM 1548a Typical Diet	1.10 ± 0.10	1.13 ± 0.09	102.5
Ni		0.37 ± 0.02	0.38 ± 0.04	102.3
Pb		0.044 ± 0.000	0.044 ± 0.013	98.9
Cd	SRM 1567a Wheat Flour	0.026 ± 0.002	0.026 ± 0.008	98.4
Co		0.006 ± 0.00	0.006 ± 0.002	102.4
Cu		2.1 ± 0.2	2.09 ± 0.4	99.7
Fe		14.1 ± 0.5	13.9 ± 0.3	98.9
Mn		9.4 ± 0.9	9.6 ± 1.5	102.4
V		0.011 ± 0.00	0.011 ± 0.00	99.4
Zn		11.6 ± 0.4	11.9 ± 0.2	102.7

R: recovery percentage.

**Table 3:** Mean concentration (mg/kg wet weight) and standards deviations (SD) for the analyzed metals according to the tissue type.

Element	Concentration (mg/kg Wet Weight) ± SD	
	Muscle Tissue	Liver Tissue
Al	1.46 ± 0.78	36.2 ± 28.9
Cd	0.007 ± 0.01	1.40 ± 1.93
Pb	0.03 ± 0.01	0.43 ± 0.59
Ba	0.72 ± 0.22	1.60 ± 2.06
B	0.19 ± 0.20	2.81 ± 5.23
Co	0.01 ± 0.01	0.42 ± 0.27
Cu	0.70 ± 0.35	47.0 ± 78.4
Cr	0.35 ± 0.68	0.18 ± 0.56
Sr	0.43 ± 1.35	3.49 ± 7.12
Fe	6.55 ± 7.57	221 ± 158
Li	0.29 ± 0.18	2.37 ± 2.03
Mn	0.42 ± 0.36	1.38 ± 1.65
Mo	0.02 ± 0.003	0.39 ± 0.43
Ni	0.12 ± 0.33	0.45 ± 0.74
V	0.11 ± 0.14	1.73 ± 2.03
Zn	16.3 ± 6.34	76.4 ± 79.6

**Table 4:** Mean concentration (mg/kg wet weight) and standard deviations (SD) of the toxic metals and trace elements in the analyzed muscle and liver tissue according to zones.

Element	Muscle Tissue Concentration (mg/kg Wet Weight) ± SD			Liver Tissue Concentration (mg/kg Wet Weight) ± SD		
	Metropolitan Zone	South Zone	North Zone	Metropolitan Zone	South Zone	North Zone
Al	1.69 ± 1.18	1.38 ± 0.44	1.31 ± 0.56	57.7 * ± 30.6	23.1 ± 19.2	27.8 ± 24.4
Cd	0.0007 * ± 0.0005	0.01 * ± 0.01	0.008 * ± 0.06	0.65 ± 0.58	0.80 ± 0.54	2.74 * ± 3.00
Pb	0.04 ± 0.01	0.03 ± 0.006	0.03 ± 0.01	0.83 * ± 0.85	0.17 * ± 0.11	0.29 * ± 0.33
Ba	0.26 ± 0.36	0.10 ± 0.04	0.16 ± 0.08	3.69 * ± 2.41	0.77 ± 0.66	0.34 ± 0.18
B	0.18 * ± 0.26	0.08 * ± 0.08	0.31 * ± 0.16	5.52 * ± 8.63	1.29 ± 0.58	1.62 ± 1.05
Co	0.01 * ± 0.005	0.004 * ± 0.006	0.02 * ± 0.02	0.43 ± 0.30	0.51 ± 0.22	0.31 ± 0.27
Cu	0.49 * ± 0.05	0.73 * ± 0.41	0.86 * ± 0.39	87.0 * ± 112	29.8 * ± 64.1	24.3 * ± 19.2
Cr	0.08 * ± 0.03	0.73 ± 0.41	0.89 ± 1.00	<LOQ **	0.42 * ± 0.94	0.12 ± 0.09
Sr	1.00 ± 2.10	<LOQ **	0.29 ± 0.92	6.45 * ± 12.0	2.79 ± 1.88	1.25 ± 0.73
Fe	2.94 * ± 0.54	4.26 * ± 1.65	12.5 * ± 11.1	301 * ± 181	136 * ± 69.0	228 * ± 166
Li	0.23 ± 0.11	0.35 ± 0.21	0.28 ± 0.20	2.05 ± 1.12	2.79 ± 3.15	2.29 ± 1.31
Mn	0.49 ± 0.57	0.28 * ± 0.14	0.48 ± 0.21	1.98 ± 2.65	1.10 ± 0.71	1.05 ± 0.82
Mo	0.04 ± 0.001	0.01 ± 0.003	0.02 ± 0.004	0.40 ± 0.42	0.27 * ± 0.16	0.50 ± 0.61
Ni	0.05 ± 0.04	0.08 ± 0.07	0.24 * ± 0.56	0.65 ± 1.24	0.50 ± 0.31	0.19 * ± 0.12
V	0.04 * ± 0.10	0.09 * ± 0.08	0.19 * ± 0.18	2.35 ± 2.14	0.61 * ± 1.08	2.38 ± 2.32
Zn	16.2 ± 4.23	17.4 ± 6.00	15.4 ± 8.56	77.9 * ± 72.0	38.2 * ± 32.1	113 * ± 105

* Statistical differences (*p* < 0.05) ** LOQ, limit of quantification

**Table 5:** Spearman correlation coefficient for Cd and Pb according to length and weight.

Spearman Correlation Coefficient	Concentration (mg/kg) in Muscle	
	Cd	Pb
Length	0.568	−0.359
Weight	0.635	−0.393

**Table 6:** Estimated daily intake values (EDI) (mg/day) and percentages of contribution to the dietary intake.

Element	Guideline Values	Parameter	Reference	EDI (mg/kg) ^a^		Contribution ^b^ (%)	
				Muscle Tissue	Liver Tissue	Muscle Tissue	Liver Tissue
Mn	-	-	-	0.04	0.14	-	-
Li				0.03	0.24		
Co				0.001	0.04		
Cu	10 mg/day	UL	[21]	0.07	4.70	0.70	47.0
Fe	45 mg/day			0.66	22.1	1.47	49.1
Mo	0.1–0.5 mg/day			0.002	0.04	2.0–0.40	40.0–8.0
Zn	40 mg/day			1.63	7.64	4.10	19.1
B	1.7–2.0 mg/day			0.02	0.28	1.18–1.00	16.5–14.0
V	1.8 mg/day			0.01	0.17	0.56	9.44
Ba	0.2 mg/kg bw/day	TDI	[24]	0.07	0.16	0.51	1.17
Sr	0.13 mg/kg bw/day		[25]	0.04	0.35	0.45	3.93
Ni	2.8 µg/kg bw/day		[28]	0.01	0.05	5.22	26.1
Cd	2.5 µg/kg bw/week	TWI	[42]	0.0007	0.14	2.86	572
Pb	0.5 µg/kg bw/day	TDI	[43]	0.003	0.04	8.76	117
Cr	0.3 mg/kg bw/day		[44]	0.04	0.02	0.19	0.10
Al	1 mg/kg bw/week	TWI	[45]	0.15	3.62	1.53	37.0

^a^ Supposing a consumption of 100 g per day of Sarpa salpa. ^b^ Mean weight of an adult of 68.48 kg [46]. Bw: body weight, UL: upper level intake, TDI: tolerable daily intake, TWI: tolerable weekly intake.
